# Clearing Traffic Jams During Protein Translocation Across Membranes

**DOI:** 10.3389/fcell.2020.610689

**Published:** 2021-01-08

**Authors:** Lihui Wang, Yihong Ye

**Affiliations:** Laboratory of Molecular Biology, National Institute of Diabetes and Digestive and Kidney Diseases, National Institutes of Health, Bethesda, MD, United States

**Keywords:** UFM1, ribosome UFMylation, ribosome-associated quality control, ribosome stalling, translocon-associated quality control, translocon clogging, protein translocation, endoplasmic reticulum

## Abstract

Protein translocation across membranes is a critical facet of protein biogenesis in compartmentalized cells as proteins synthesized in the cytoplasm often need to traverse across lipid bilayers *via* proteinaceous channels to reach their final destinations. It is well established that protein biogenesis is tightly linked to various protein quality control processes, which monitor errors in protein folding, modification, and localization. However, little is known about how cells cope with translocation defective polypeptides that clog translocation channels (translocons) during protein translocation. This review summarizes recent studies, which collectively reveal a set of translocon-associated quality control strategies for eliminating polypeptides stuck in protein-conducting channels in the endoplasmic reticulum and mitochondria.

## Introduction

The endoplasmic reticulum (ER) is the entry point of the secretory pathway in eukaryotic cells: Most proteins destined for the endomembrane system or extracellular space are first inserted into the ER membrane or translocated into the ER lumen (Fewell and Brodsky, 2009; Zimmermann et al., 2011). These proteins are folded, assembled, and modified in the ER before reaching their final destinations (Fewell and Brodsky, 2009). Approximately one-third of the eukaryotic proteome is processed in the ER (Chen et al., 2005). Most of these proteins are imported into the ER by the Sec61 translocon *via* either a cotranslational or posttranslational targeting mechanism.

The cotranslational translocation pathway moves nascent polypeptides into the ER while they are being synthesized by ER-bound ribosomes. This process is used by ER clients bearing an amino-terminal signal sequence or a hydrophobic transmembrane domain (TMD), which is recognized by signal recognition particle (SRP) in the cytosol. SRP then targets the ribosome-nascent chain complex to the ER membrane *via* an ER-localized SRP receptor. Nascent chains are handed over to the Sec61 translocon, which uses the energy from translating ribosomes to move polypeptides across the membrane. For membrane proteins, the translocation process is coupled to membrane integration of hydrophobic TMDs through a lateral gate of the translocon (Zimmermann et al., 2011). For a subset of polytopic membrane proteins such as G protein–coupled receptors, the proper engagement of the N-terminal TMD with the translocon and lipid bilayer requires an additional complex named ER membrane protein complex (EMC) (Chitwood et al., 2018; Guna et al., 2018; Chitwood and Hegde, 2019).

A relatively small number of proteins such as those bearing a suboptimal signal sequence or the so-called tail-anchored (TA) proteins (carrying a single membrane targeting TMD at the carboxyl-terminus) use a posttranslational mechanism for ER targeting (Zimmermann et al., 2011; Ast et al., 2013). In this case, nascent polypeptides are completely synthesized in the cytosol prior to ER targeting. Substrates are kept in an unfolded, translocation-competent state by association with cytosolic chaperons (e.g., Hsp70 or the Bag6-SGTA holdase system) (Ngosuwan et al., 2003; Johnson et al., 2013). For proteins bearing a suboptimal signal sequence, they use a posttranslational translocation machinery containing the accessory proteins Sec62 and Sec63 in addition to the Sec61 translocon (Johnson et al., 2013; Gemmer and Forster, 2020). Recent cryo–electron microscopy (cryo-EM) studies showed that the binding of the Sec62-Sec63 subcomplex to the translocon induces the opening of the lateral gate, priming the channel for insertion of low hydrophobic signal sequences (Itskanov and Park, 2019; Wu X. et al., 2019). While signal sequence is moved into the lateral gate, chaperones dissociate from the remaining polypeptide, which is then threaded into the lumen by luminal ATPase BiP (Matlack et al., 1999; Itskanov and Park, 2019; Wu X. et al., 2019). For TA proteins, Bag6 and SGTA form a chaperone complex, transferring them to a downstream ATPase named TRC40 for membrane insertion (Chio et al., 2017).

## The Diverse Protein Quality Control Systems at the ER

The biogenesis of membrane and secretory proteins is monitored by various protein quality control (PQC) mechanisms, which safeguard defects in protein folding, assembly, and localization. Deficiencies in ER-associated PQC result in accumulation of malfunctional proteins and trigger ER stress, which contribute to the pathogenesis of many human diseases (Lindholm et al., 2006; Lin et al., 2008; Ozcan and Tabas, 2012).

**FIGURE 1:**
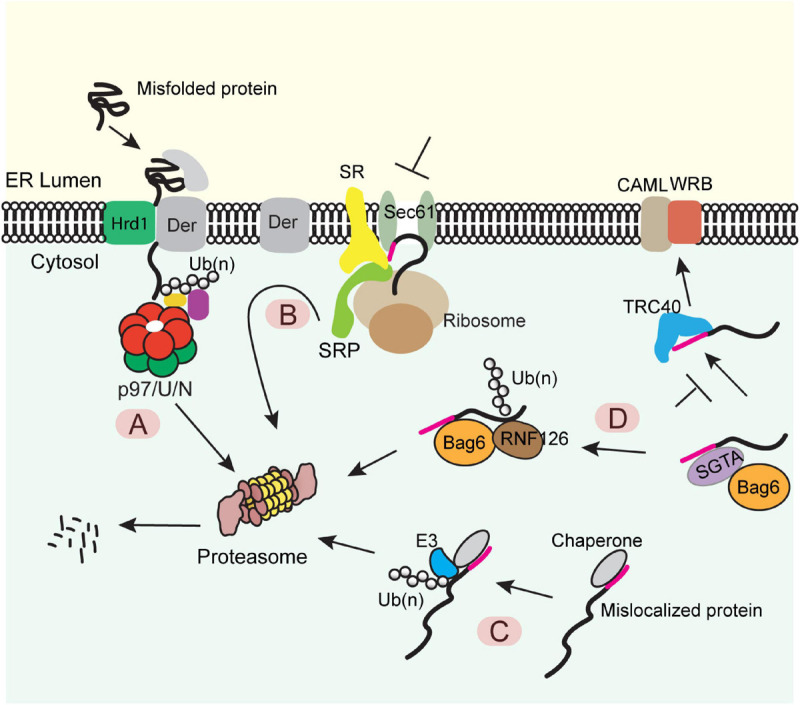
Diverse protein quality control (PQC) mechanisms at the ER membrane **(A)** The ERAD pathway. Misfolded proteins of the ER were chaperoned to a retrotranslocation complex consisting of the membrane-associated ubiquitin ligase Hrd1 and a multitransmembrane protein Derlin (Der) (note that other accessory proteins are not shown). Substrate was retrotranslocated, polyubiquitinated (Ub) by Hrd1, extracted from the membrane by the p97 ATPase and its cofactors Ufd1-Npl4 (U/N), and degraded by the proteasome. **(B)** The pre-emptive quality control pathway. Under ER stress, the SRP-dependent cotranslational translocation was attenuated. The ER-targeted nascent chains are rerouted to the cytosol for proteasome degradation. SR, SRP receptor. **(C)** PQC for mislocalized ER proteins bearing a signal sequence or a TMD. ER proteins mislocalized in the cytosol are identified by cytosolic chaperons, polyubiquitinated by an associated ubiquitin ligase and degraded by the proteasome. **(D)** A triaging pathway for mislocalized TA proteins. The engagement of a TA substrate–SGTA complex with the targeting factor TRC40 results in a quick transfer of the client protein to TRC40, which further targets the TA protein to the WRB-CAML complex for membrane insertion. When this targeting process fails, TA protein can be transferred from SGTA to BAG6. The latter recruits the ubiquitin ligase RNF126, which ubiquitinates the substrate for proteasome degradation.

The best studied ER PQC process is the ER-associated protein degradation (ERAD) pathway (Figure 1A). In this pathway, misfolded or misassembled proteins are moved back into the cytoplasm *via* a process termed *retrotranslocation*, which requires a membrane complex and the associated ubiquitination machinery. Polypeptides are subsequently extracted from the membrane by an abundant ATPase complex named Cdc48 in yeast or p97 in mammals (Christianson and Ye, 2014; Ruggiano et al., 2014; Bhattacharya and Qi, 2019). This ATPase complex acts with a few proteasome-associated chaperones such as Bag6 and ubiquilins to transfer the extracted polypeptides to the proteasome for degradation (Lim et al., 2009; Claessen and Ploegh, 2011; Wang et al., 2011). Genetic and proteomic studies have identified most ERAD components (Hampton et al., 1996; Knop et al., 1996; Christianson et al., 2011; Leto et al., 2019), while recent cryo-EM studies have revealed significant mechanistic details on how misfolded proteins are retrotranslocated by the Hrd1 ubiquitin ligase complex (Schoebel et al., 2017; Wu et al., 2020).

When the folding capacity of the ER is mitigated during ER stress, cells can initiate a preemptive quality control (ER-pQC) pathway (Kang et al., 2006; Swanton and High, 2006), which attenuates protein translocation while routing incompletely targeted proteins for degradation in the cytosol (Figure 1B) (Kang et al., 2006; Kadowaki et al., 2015). Interestingly, the ER-pQC appears to use an ERAD-like mechanism to dispose of proteins bearing certain types of signal sequences (Kang et al., 2006) as it requires the ERAD components Derlins, p97, Hrd1, and Bag6 (Kadowaki et al., 2015, 2018). The current model suggests that a Derlin family member captures translocation-attenuated nascent chains *via* interactions with SRP and an SRP receptor and reroutes them to an Hrd1-containing retrotranslocon for ubiquitination-mediated degradation (Figure 1B).

Protein quality control also eliminates polypeptides mislocalized due to errors in ER targeting. For proteins bearing a signal sequence or TMDs, the exposure of these hydrophobic elements in the cytosol generates a “degron,” causing their degradation by the ubiquitin proteasome system *via* a mechanism similar to the well-established cytosolic PQC system (Figure 1C) (Buchberger et al., 2010; Chen et al., 2011). For TA proteins mislocalized in the cytosol, prolonged association with the targeting chaperone Bag6 converts Bag6 from a targeting factor to a degradation triaging promoter as it recruits the ubiquitin ligase RNF126 to ubiquitinate the associated TA proteins (Figure 1D) (Rodrigo-Brenni et al., 2014).

Although the concept of ER PQC is well established, and many ER PQC processes have been the subject of extensive studies, previous efforts have mainly focused on folding deficiencies either after or prior to ER targeting. It was only until recently that researchers began to appreciate the error-prone facet of membrane translocation: When protein translocation is erroneously halted, the partially translocated polypeptides clog the translocon and disrupt ER homeostasis. How cells cope with clogged translocons has emerged as an intriguing question. Conceptually, several previously established PQC mechanisms could be adopted to resolve a jammed translocon. For instance, a clogged translocon may be viewed as a defective membrane complex and thus might be disposed of by ERAD, which is known for its capacity to degrade misassembled translocon components (Biederer et al., 1996; Needham and Brodsky, 2013). Alternatively, an ERphagy mechanism could allow autophagosomes to engulf damaged ER that contains clogged translocons (Strzyz, 2020). However, recent studies suggest several previously unappreciated translocon-associated quality control (TAQC) strategies that safeguard protein translocation by selectively removing stalled polypeptides.

## Translocon Declogging During Posttranslational Translocation at the ER

The Sec61 translocon is a highly conserved trimeric complex consisted of Sec61α, Sec61β, and Sec61γ in mammals or Sec61YEG in bacteria. It forms a narrow conduit, accommodating only unfolded linear polypeptides during translocation (Van den Berg et al., 2004). Therefore, translocation clogging could occur during posttranslational translocation if a yet-to-be translocated domain has become folded prior to translocation. This could result from either chaperone deficiency or premature dissociation of chaperones from polypeptides undergoing translocation.

**FIGURE 2:**
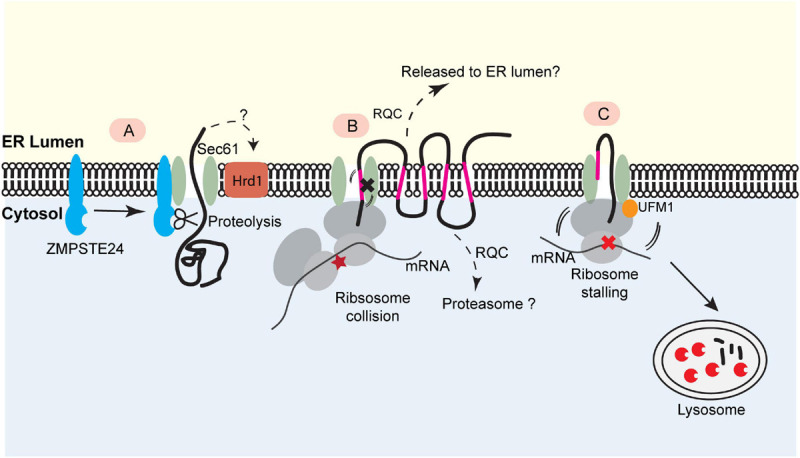
Translocon-associated quality control at the ER. **(A)** For posttranslational protein translocation, translocon clogging occurs when the Sec61 translocon engages a client protein with a folded domain. This causes the recruitment of the ER- bound protease ZMPSTE24 (Ste24 in yeast), which cleaves the protein in the translocon for further degradation. This process may also involve Hrd1. **(B)** The biogenesis of polytopic membrane proteins depends on cotranslational insertion of TMDs into the ER membrane. A defect in TMD membrane integration can stall translocation process, causing ribosome collision. This may trigger a mechanism analogous to ribosome-associated quality control (RQC) to eliminate the faulty nascent proteins. The precise degradation mechanism for stalled membrane proteins (e.g., released into the ER lumen or extracted into the cytosol) is not clear. **(C)** Ribosome UFMylation-dependent trafficking of stalled nascent chains to lysosome. Ribosome stalling during cotranslational protein translocation induces UFMylation of the 60S ribosomal subunit RPL26, which allows the trafficking of stalled nascent chains to lysosomes for degradation.

Ast et al. (2016) recently identified the yeast metalloprotease Ste24 (or ZMPSTE24 in mammals) as a key quality control factor that resolves clogged translocons during posttranslational translocation (Figure 2A). Ste24/ZMPSTE24 is an integral membrane protein originally reported as a zinc metalloprotease responsible for the cleavage of prenylated (farnesylated or geranylgeranylated) substrates (Goblirsch and Wiener, 2020). It also processes lamin A precursor to facilitate its maturation (Kilic et al., 1997). To explore how yeast cells cope with “translocon clogging” during posttranslational translocation, Ast and colleagues designed a model substrate containing a rapidly folding, tightly packed domain. They found that the folding of this substrate prior to ER translocation causes translocon clogging, resulting in the recruitment of Ste24 (ZMPSTE24 in mammals) to the clogged translocon and subsequent cleavage of the partially translocated polypeptide by the metalloprotease activity of Ste24 (Ast et al., 2016). Besides Ste24, a recent study demonstrated a role for Hrd1 in degradation of this model substrate, probably *via* an ERAD-like or the aforementioned ER-pQC mechanism (Runnebohm et al., 2020). These mechanisms may collectively resolve clogged translocons to maintain the secretory homeostasis (Figure 2A).

Although Ste24 was suggested as a translocon “unclogger” specifically for posttranslational translocation, a more recent genetic screen in *Saccharomyces cerevisiae* identified it as a suppressor of proteotoxicity induced by amyloid polypeptide (IAPP). IAPP is a signal sequence-containing secretory protein that can clog the translocon by induced oligomerization prior to ER targeting. IAPP oligomerization-associated cytotoxicity in pancreatic β cells is considered a key contributor to type 2 diabetes. Overexpression of an oligomerization-prone IAAP variant in yeast also causes cytotoxicity, which was attributable to a translocation defect because the suppressor function of Ste24 depends on its declogging activity (Kayatekin et al., 2018). Although it is unclear why IAPP is poorly translocated even with an efficient signal sequence, this study underscores a potential detrimental consequence of obstructing ER translocon, which may define a new pathological hallmark for secretory pathway-associated amyloid-like diseases.

How does Ste24 sense translocon clogging? Conceptually, in a clogged translocon, substrate likely engages the translocon in an erroneous way, which may leave a conformational “mark” for Ste24 recognition. Consistent with this hypothesis, a recent study showed that in budding yeast, Ste24 prevents inappropriate engagement of the translocon with substrates lacking signal sequence, which may otherwise generate a clogging-like state (Hosomi et al., 2020).

## Translocon Declogging During Cotranslational Translocation

### Scenarios of Translocon Clogging During Cotranslational Protein Translocation

In cotranslational protein translocation, polypeptides are pushed into the Sec61 translocon in a linear way by translating ribosome docked on the translocon. Only until the nascent chain is fully integrated into the ER does the ribosome dissociate from the membrane, allowing downstream ribosomes to engage the translocon for subsequent rounds of protein translocation. As protein translocation is tightly coupled to translation, protein folding, membrane insertion, and protein modifications, this coordinated process can be perturbed by either ribosome stalling or defects in folding, protein modification, and membrane insertion.

Translation of faulty mRNAs bearing no stop codon (NS mRNAs) represents a major trigger of ribosome stalling both in the cytosol and on the ER membrane (Brandman and Hegde, 2016; Joazeiro, 2017, 2019; Sitron and Brandman, 2020). NS mRNAs are frequently generated in eukaryotic cells due to genetic mutations or premature polyadenylation (Arthur et al., 2015), resulting in a poly-A–containing open reading frame that can stall ribosomes during translation (Arthur et al., 2015; Juszkiewicz and Hegde, 2017). Faulty mRNAs bound to the ER may also be generated during ER stress. It is well appreciated that ER stress–activated inositol-requiring enzyme 1 (Ire1) contains both a kinase and an endonuclease activity. The latter can cleave mRNAs encoding membrane and secretory proteins *via* a process dubbed as regulated Ire1-dependent mRNA decay (RIDD) (Hollien and Weissman, 2006; Hollien et al., 2009). This process attenuates the protein flux into the ER to mitigate ER stress, but some truncated mRNAs lacking stop codon might be generated by RIDD, which could cause ribosome stalling.

Unlike ribosome stalling in the cytosol, translation arrest during cotranslational translocation generates a unique proteostasis crisis because the arrested products are embedded in the Sec61 translocon: A significant portion of the substrate upstream of the stalling sequence is either in the ER lumen or has been integrated into the ER membrane and thus could have participated in cotranslational events such as protein folding, glycosylation, and disulfide bond formation. Consequently, translation arrests not only produce defective nascent polypeptides, but also generate clogged translocons and a blockade in ER import (Izawa et al., 2012; Arakawa et al., 2016). Apparently, stalled ribosomes must be released from the membrane, so the aberrant translocation products can be eliminated.

The Sec61 translocon can also be clogged when a polytopic membrane protein fails to be inserted into the membrane due to defects in TMD specific chaperones. The biogenesis of polytopic membrane proteins can pose a significant challenge to cells because when an amino-terminal amphipathic TMD exits the translocon *via* the lateral gate, it often needs to wait in the hydrophobic lipid environment for assembly with downstream TMDs *via* non-hydrophobic interactions. Recent studies have identified the EMC as a potential TMD-specific chaperone (Chitwood et al., 2018; Guna et al., 2018; Chitwood and Hegde, 2019). Accordingly, defects in EMC-mediated TMD membrane integration are expected to stall the translocation process and cause “translocon clogging.”

### Ribosome Stalling and Ribosome-Associated Quality Control

Studies on ribosome stalling in the cytosol have established a PQC pathway named ribosome-associated quality control (RQC) (Brandman and Hegde, 2016; Joazeiro, 2017; Sitron and Brandman, 2020). In this process, a collection of cellular factors coordinately sense translation stalling, rescue stalled ribosomes, and degrade arrested polypeptides together with defective mRNAs. Specifically, when a translating ribosome stalls on an mRNA, the following ribosome will collide with the stalled ribosome given the polysome nature of protein translation. This generated a unique “disome” signature (Juszkiewicz and Hegde, 2017; Juszkiewicz et al., 2018; Ikeuchi et al., 2019), which is sensed by the ribosome-associated ubiquitin ligase ZNF598 (hel2 in yeast). ZNF598 ubiquitinates ribosomes at several sites to initiate Dom34/Hbs1-mediated disassembly of ribosomes (Juszkiewicz and Hegde, 2017; Matsuo et al., 2017; Sundaramoorthy et al., 2017), generating a 60S-peptidyl-tRNA subcomplex that further engages downstream RQC factors (Juszkiewicz et al., 2020; Matsuo et al., 2020). Eventually, the linked peptidyl-tRNA was cleaved off from nascent chains by an endonuclease (e.g., ANKZF1) (Kuroha et al., 2018; Verma et al., 2018; Zurita Rendon et al., 2018). The arrested nascent chains are then ubiquitinated by the 60S ribosome-associated ubiquitin ligase Listerin (Ltn1 in yeast) (Bengtson and Joazeiro, 2010), extracted from the ribosome by the Cdc48/p97 ATPase complex and degraded by the proteasome (Brandman and Hegde, 2016; Joazeiro, 2017).

In sharp contrast to the well-documented RQC mechanism, the limited information regarding ribosome stalling on mRNAs encoding secretory or membrane proteins fails to paint a consistent model. Izawa et al. (2012) initially showed that an ER-targeted model substrate bearing no stop codon could be released into the ER lumen following Dom34/Hbs1-mediated ribosome dissociation and that the released substrates appear to escape degradation by the proteasome. However, subsequent studies in yeast suggested that Ltn1-mediated ubiquitination and proteasomal degradation contribute to the cotranslational degradation of arrested secretory and membrane proteins produced by ribosome stalling on the ER membrane (Crowder et al., 2015; Arakawa et al., 2016). Likewise, a recent study showed an ERAD-like mechanism that degrades a translation-arrested product at the ER in mammalian cells (Cesaratto et al., 2019). Furthermore, biochemical fractionation revealed the recruitment of RQC factors such as Listerin and NEMF to the ER membrane in response to ribosome stalling in a cell-free system (von der Malsburg et al., 2015), but given that translocating nascent chains are usually shielded by the translocon and the associated ribosome, the study did not reveal how arrested products could be recognized by Listerin on the cytosolic side of the ER membrane.

The aforementioned contradiction may be resolved if cotranslational TAQC mechanisms are substrate-tailored (Figures 2B,C). A conceivable distinction between different TAQC substrates may lie in the state of translocon clogging. Transient clogging due to suboptimal signal sequence may result in the recruitment of ER chaperons to resolve clogged translocon while keeping the nascent chains in the biosynthetic path (Sha Sun, 2020), but aberrant translocon engagement for substrates with either a translocation-impeding domain or deregulated lipid binding (e.g., apoB) may reverse the translocation process, causing the degradation of aberrant polypeptides in the cytosol (Hrizo et al., 2007; Rubenstein et al., 2012; Runnebohm et al., 2020). By contrast, permanent stalling by translation arrest might lock the translocation process in an irreversible state, prompting the release of the stalled polypeptides only toward the ER lumen for clearance by other mechanisms (see below).

### TAQC During Polytopic Membrane Protein Biogenesis

In yeast mutants lacking a functional EMC, the biosynthesis of a mutant ABC transporter Yor1ΔF is compromised, which appears to cause ribosome stalling (Figure 2B). Lakshminarayan et al. (2020) suggested that when defects in TMD integration arise, the activation of RQC contributes to the translation arresting of Yor1ΔF. In accordance with this interpretation, reducing translation initiation or knockout of upstream RQC factors could partially rescue the biogenesis defects of Yor1ΔF, probably because these perturbations lower the ribosome density on the Yor1ΔF mRNA to avoid ribosome collision and subsequent RQC activation (Lakshminarayan et al., 2020). Additionally, translatome-wide ribosome profiling revealed that polytopic membrane proteins generally have a low translation efficiency in yeast, suggesting an evolution pressure to keep RQC in check in polytopic membrane proteins biogenesis (Lakshminarayan et al., 2020). Another study in human cells identified several multitransmembrane proteins as potential RQC substrates because their degradation is partially mediated by Listerin (Trentini et al., 2020). Although these studies suggested a link between translocon clogging and certain components of the RQC pathway, the precise fate of the arrested translocation products was not explored. In this regard, a systemic analysis of the involvement of the known cytosolic RQC factors in the degradation of stalled polytopic membrane proteins is warranted. Moreover, the biogenesis of soluble secretory proteins or membrane proteins with low numbers of TMDs does not seem to follow the aforementioned quality control mechanism (Trentini et al., 2020), suggesting the existence of additional mechanism(s) that declogs the translocon during cotranslational ER targeting.

### TAQC for Nascent Chains Bearing Ribosome Stalling Sequences

To study how mammalian cells cope with ribosome stalling during cotranslational translocation, we recently generated an ER stalling reporter containing a polyadenine segment downstream of an ER targeting signal sequence. The reporter also contains a GFP-encoding sequence upstream of the stalling site and an RFP-coding sequence downstream. Translation stalling results in a translocon-associated translation product bearing GFP but no RFP. As expected, this translocon-clogging nascent chain is short-lived, but surprisingly, it is not degraded by the proteasome. Instead, it is transported out of the ER and disposed of by lysosomes. Intriguingly, this process requires the modification of the ribosome subunit RPL26 with a ubiquitin-like molecule named UFM1 (ubiquitin-like modifier 1) (Figure 2C) (Wang et al., 2020).

Protein modification by UFM1, a process dubbed as UFMylation is a conserved posttranslational modification found in most eukaryotes except in yeast and fungi (Cappadocia and Lima, 2018; Gerakis et al., 2019). In analogous to ubiquitination, UFMylation is mediated by an enzyme cascade involving UBA5 as the activating enzyme (E1), UFC1 as the conjugating enzyme (E2), and an ER-localized trimeric ligase (E3) complex composed of DDRGK1 (also named UFBP1), UFL1, and Cdk5RAP3. The UFMylation system has been tightly linked to ER protein homeostasis: the expression of most UFMylation enzymes is upregulated by ER stress, and conversely, deficiencies in UFMylation enzymes are known to sensitize cells to ER stress–induced apoptosis (Komatsu et al., 2004; Lemaire et al., 2011; Zhang et al., 2015; Wei and Xu, 2016). However, the UFMylation substrate (s) accountable for the deregulation of ER homeostasis in UFMylation-deficient cells has been elusive.

We and other recently identified the 60S ribosomal subunit RPL26 (ul24) as the principal target of UFMylation (Walczak et al., 2019; Wang et al., 2020). Biochemical fractionation demonstrated that UFM1 preferentially modifies ER-bound ribosomes, consistent with the ER localization of the UFM1 ligase complex. UFMylation occurs in two conserved lysine residues (K132 and K134 in human RPL26) in a short C-terminal tail, which appears to be co-evolved with the UFMylation system (Walczak et al., 2019; Wang et al., 2020). Importantly, we showed that translation stalling during cotranslational ER translocation is a specific trigger for ribosome UFMylation. This finding links ribosome UFMylation to TAQC. In support of this notion, we showed that RPL26 UFMylation promotes the ER exit and lysosomal transport of an arrested nascent chain reporter: In cells lacking either the UFMylation system or the modification sites on endogenous RPL26, the polypeptides stalled in the translocon are cleared at a reduced rate, which disrupts ER homeostasis (Wang et al., 2020).

The physiological substrates subjected to UFM1-dependent clearance are currently unknown, but several recent studies echo our findings by demonstrating the requirement of the UFMylation system for lysosomal transport of other defective quality control substrates including an ERphagy reporter (Liang et al., 2020; Stephani et al., 2020), suggesting that UFMylation may regulate the trafficking of a spectrum of defective proteins broader than initially thought. Genetic studies in mice have revealed an indispensable function of UFMylation in cell differentiation and animal development (Cai et al., 2016, 2019; Zhu et al., 2019). Knockout of UFMylation E1 or E3 leads to a severe defect in erythroid lineage differentiation, causing anemia in animals (Tatsumi et al., 2011; Cai et al., 2015). Consistently, in an *in vitro* erythropoiesis model, RPL26 UFMylation is upregulated during erythroid differentiation, which coincides with an increase in the secretory flow. Compromising ribosome UFMylation impairs protein secretion, induces ER stress, and ultimately inhibits hemoglobin production (Wang et al., 2020). Along a similar line, DDRGK1, a key component of the UFM1 ligase complex appears to play a crucial role in the development of antibody-secreting plasma cells (Zhu et al., 2019). These results collectively establish a physiological link between ribosome UFMylation and secretory homeostasis *via* UFMylation dependent TAQC. Intriguingly, genetic mutations in genes encoding UFMylation enzymes are linked to a variety of neurological disorders in humans (Colin et al., 2016; Muona et al., 2016; Nahorski et al., 2018). Whether defective TAQC underlies the molecular driver of these diseases remains to be investigated (Colin et al., 2016; Muona et al., 2016; Mignon-Ravix et al., 2018; Nahorski et al., 2018).

## TAQC at the Mitochondria

In eukaryotes, protein translocation machinery is present in a variety of organelles/membranes besides the ER, raising the possibility of additional TAQC pathways in other protein translocation systems. Indeed, recent studies have shed light on several quality surveillance mechanisms that clear stalled precursor proteins from the mitochondrial outer membrane (MOM).

The mitochondrial protein translocation system consists of the Tom20 complex in the outer membrane and two Tim complexes in the inner membrane (Tim22 and Tim23), which import mitochondrial proteins synthesized in the cytosol (Wiedemann et al., 2004; Wickner and Schekman, 2005). The mitochondrial protein translocation-associated degradation (MitoTAD) pathway was recently reported in *S. cerevisiae*, which constantly monitors the TOM machinery under non-stress conditions to prevent channel clogging (Martensson et al., 2019). A key player is a transmembrane protein named Ubx2, which was previously implicated in ERAD as a membrane adaptor for Cdc48. However, Martensson et al. (2019) showed that a pool of Ubx2 associates with the Tom20 translocase and recruits Cdc48 to remove translocation-arrested precursor proteins. The mitoCPR (mitochondrial compromised protein import response) pathway is another mechanism activated by the stalling of mitochondrial precursor proteins in the Tom translocon, but it primarily acts during stress. Weidberg et al. showed that overexpression of a bipartite signal–containing mitochondrial protein in yeast clogs the Tom translocon, which in turn induces the expression of Cis1 through a transcription factor named Pdr3. Cis1 associates with the clogged Tom translocon, recruiting another ATPase named Msp1 to extract stalled precursor proteins for proteasome degradation (Weidberg and Amon, 2018).

Both the MitoTAD and MitoCPR mechanisms appear to cope with polypeptides stalled during posttranslational translocation, which accounts for most translocation events at the mitochondria. However, recent studies also suggest the existence of localized protein translation in proximity to the MOM, which may result in cotranslational protein import into the mitochondria (Williams et al., 2014; Lesnik et al., 2015). Accordingly, mitochondrial stress due to the loss of membrane potential, etc., can cause ribosome stalling when polypeptides undergo cotranslational translocation *via* the Tom translocon (Wu et al., 2018). This defect activates a quality control mechanism. Specifically, Ltn1 was recruited to stalled nascent chains to facilitate their degradation (Izawa et al., 2017). Intriguingly, stalled nascent chains are released into the mitochondria matrix by Dom34/Ski7 and Vms1 and degraded by matrix resident proteases (Izawa et al., 2012, 2017). This mechanism resembles UFM1-dependent declogging at the ER, which also releases nascent chains into the lumen for transport to lysosomes. Defects in mitochondria-associated TAQC result in protein aggregates in the mitochondria, which sequesters chaperones and proteases to cause cytotoxicity (Izawa et al., 2017; Wu Z. et al., 2019).

Translocation clogging at the MOM might also induce mitophagy, a form of macroautophagy that selectively removes damaged mitochondria to resolve translocon jamming. Wu et al. (2018) showed that loss of MOM potential induces PINK1-dependent recruitment of a ribosome-nascent chain complex containing the mRNA encoding the 30-kD complex-I subunit (c-I30) (Gehrke et al., 2015). Ribosome stalling on c-I30-encoding mRNA triggers the recruitment of RQC factors such as Pelo, ABCE1, and NOT4. As a result of polyubiquitination of ABCE1 by NOT4, mitophagy is initiated, which clears damaged mitochondria including clogged translocons (Wu et al., 2018). Collectively, these studies suggest the use of diverse TAQC strategies to resolve translocon clogging by mitochondria.

## Perspectives

Protein translocation across membranes is critical for cell physiology because it is a fundamental protein biogenesis step for clients destined to secretion or the endomembrane system. While the translocation machineries in different organelles have been extensively studied, the quality control mechanisms dedicated to the surveillance of these translocation processes have just begun to surface. Although TAQC is now being characterized in the ER and mitochondria, similar quality control processes may exist in other organelles such as peroxisomes and chloroplasts.

As a newly emerged field, there are many outstanding questions to be explored for TAQC. What are the physiological substrates of the each TAQC pathway? Are these TAQC mechanisms regulated by stress or under aging conditions in animals? How do cells sense clogged translocons in different TAQC pathways? What are the fates of the clogged substrates in each pathway? More importantly, as TAQC mechanisms are essential for the normal flux of proteins during biogenesis, the declogging function of TAQC may be particularly important for specialized cells with a high demand for protein translocation. Under these circumstances, failure in TAQC may contribute to the pathogenesis of human diseases. For instance, it would be interesting to investigate whether UFMylation-mediated TAQC plays a role in neuronal development and whether the pleiotropic neurological disorders associated with UFMylation deficiencies are caused by defects in TAQC. In short, future studies along these directions will surely reveal the full biological scope for these new PQC pathways, and also shed important insights on various pathophysiological TAQC conditions that are intimately associated with human diseases.

## Author Contributions

LW and YY conceived the topic and wrote the manuscript together. Both authors contributed to the article and approved the submitted version.

## Conflict of Interest

The authors declare that the research was conducted in the absence of any commercial or financial relationships that could be construed as a potential conflict of interest.
